# Sedentary behaviour surveillance in Canada: trends, challenges and lessons learned

**DOI:** 10.1186/s12966-020-00925-8

**Published:** 2020-03-10

**Authors:** Stephanie A. Prince, Alexandria Melvin, Karen C. Roberts, Gregory P. Butler, Wendy Thompson

**Affiliations:** 1 Centre for Surveillance and Applied Research, Public Health Agency of Canada, 785 Carling Avenue, Ottawa, Ontario K1A 0K9 Canada; 2 Division of Cardiac Prevention and Rehabilitation, University of Ottawa Heart Institute, 40 Ruskin Street, Ottawa, Ontario K1Y 4W7 Canada

**Keywords:** Sedentary behaviour, Surveillance, Population health, Measurement, Questionnaires, Accelerometers

## Abstract

**Background:**

Historical changes in the nature of sedentary activities have been observed in other countries, but it is not clear if similar trends exist in Canada. It is also unclear how changes in the measurement of sedentary behaviour affects national estimates. Our objective is to document all sources and measures of sedentary behaviour from Canadian, nationally representative surveys, and report on selected estimates of time spent in sedentary activities. Lessons learned can benefit the wider international surveillance community.

**Methods:**

We describe and document all data sources of sedentary behaviour at the national level in Canada, and report on selected prevalence data from repeated cross-sectional surveys. We summarize amounts of total device-assessed sedentary time and self-reported sedentary activities (e.g., passive travel, leisure television, computer, video games, screen, and reading) by age group over time.

**Results:**

Nineteen national surveys were identified. Changes in questions and/or response categories precluded direct assessment of trends over time for some measures; however, certain trends were observed. Accelerometer-measured sedentary time, leisure reading (among those < 50 years) and television/video viewing in younger age groups have remained relatively stable (with a possible slight decline in television/video viewing). Time spent in passive travel and leisure computer and electronic device use appears to have increased. Television and video viewing appears to have increased in older adults while their leisure reading appears to have fallen.

**Conclusions:**

Changes in measurement of sedentary behaviour can affect estimates and reduce comparability over time. Total leisure screen use appears to have increased over time, reflecting the ways in which Canadians spend their free time and technological advances. The main public health message is the need for continued efforts to reduce leisure screen use, especially among youth and older adults.

## Background

Canadians spend the majority of their days engaged in sedentary behaviour [1]. Sedentary behaviour includes activities undertaken at a low energy expenditure (≤ 1.5 metabolic equivalents) while sitting, lying or reclining, such as watching television, using a computer or sitting in a vehicle [2]. Systematic review evidence has identified that sedentary behaviour is a risk factor for several chronic conditions (e.g., cardiovascular disease, diabetes, obesity, cancer, and depression) and mortality [3–5]. Large doses of physical activity (~ 60–75 min/day) have been shown to offset the increased risk associated with prolonged sedentary behaviour [6]; unfortunately, the majority of Canadians fall well below these levels [7]. As a result, public health messaging emphasizes the importance of reducing prolonged time spent sedentary in addition to getting adequate physical activity and sleep [8]. The Canadian 24-Hour Movement Guidelines for Children and Youth include a recommendation to reduce leisure screen time to 2 h or less per day and to limit prolonged sitting [9]; guidelines for adults are under development.

The field of sedentary behaviour research is growing at a rapid rate [10], but prior to this interest, Canada had a long-standing record of measuring this behaviour. The 1985 Canada Fitness Survey asked respondents to self-report their daily sitting time; this data was subsequently used in one of the earliest papers to document the link between prolonged sitting and mortality risk [11]. Additionally, several national surveys have included questions related to screen-time; a key sedentary behaviour. The growing public health need to monitor and report on sedentary behaviour and related factors led to a modernization of the Public Health Agency of Canada’s (PHAC’s) physical activity surveillance system which is now directed by the Physical Activity, Sedentary Behaviour and Sleep (PASS) Indicators [12, 13].

Within the PASS Indicators, the PHAC reports on the sedentary behaviour levels of Canadians using the most recent and comprehensive data available [7]. While changes to survey content have permitted updates to measures/indicators to keep up with the sedentary practices of Canadians (e.g. types of screens used) [14], this has created challenges in obtaining a consistent time series of data. It is important to understand these measures in their historical context and whether trends in the types and duration of sedentary behaviour can be detected. Lessons learned can be applied in international comparisons and for the interpretation of trends in other countries.

Research in other high-income countries suggests that leisure time spent sedentary has remained fairly stable with slight variations over time [15–18]. However, the nature of leisure sedentary activities is in flux with a greater uptake of screen-based technologies beyond simply watching television [15, 16, 19–21]. Sedentary transport has also increased, albeit at a slower rate than screen use [15, 16], while other sedentary activities (e.g., socialization and crafts) have declined [16]. It is not clear if similar trends exist in Canada. The objective of this research was to document all sources and measures of sedentary behaviour from Canadian, nationally representative surveys, and report on selected data from those which are ongoing and provide estimates of time spent in various sedentary activities. We also hoped by examining how measurement has changed over time we could provide insights regarding comparability within and across countries and lessons learned for future surveillance of sedentary behaviour.

## Methods

We describe and document all sources and measures of sedentary behaviour at the national level in Canada, and report on selected prevalence data from common surveillance health surveys.

### Study population

We included results from nationally representative repeated cross-sectional surveys that assessed the sedentary behaviour of children (6–11 years), youth (12–17 years), adults (≥ 18 years), and older adults (≥ 65 years). We further divided adults into 18–34 year olds, 35–49 year olds, and 50–64 year olds.

### Measures of sedentary behaviour

Any question(s)/item(s) related to sedentary behaviour were eligible for inclusion. Questions could comprise any domain (e.g., occupational, leisure, transportation, household) or type of activity (e.g., screen time, reading, hobbies, car travel). Both device-assessed and self-report measures were included.

### Data collection procedures

An environmental scan of all national survey sources in Canada was carried out to identify measures of sedentary behaviour and an inventory created. The TAxonomy for Self-reported Sedentary behaviour Tools (TASST) framework [22] was used to describe the characteristics of identified survey questions including collection years, age of respondents, method of administration, sedentary behaviour type (e.g., total sitting, leisure television), number of questions, recall period (e.g., past 7 days), temporal unit (e.g., single day, week), assessment period (e.g., weekday only, weekend day only, total week), and response options (categorical or continuous). Table 1 includes the questions and response content for all surveys and highlights changes between years.

The prevalence of sedentary activities across survey years by age group was examined within four surveys: Canadian Community Health Survey (CCHS); Canadian Health Measures Survey (CHMS); General Social Survey (GSS); and, the Health Behaviour in School-Aged Children (HBSC) study. These four surveys were chosen as they provide ongoing, national estimates of time spent in various sedentary activities.

**Table 1:** National Canadian surveys with questions on sedentary behaviour

Survey name	Year(s)	Ages	Method of administration	Type(s) of sedentary behaviour	# of questions	Recall period, temporal unit	Response option
Canada Health Survey	1978–79	All ages	Interviewer-administered in-person without computer-assisted interviewing	Occupational (usual work activity)	1	Usual day, single day	Categorical (including “I am usually sitting during the day and do not walk very much”)
Canada Fitness Survey	1981	≥ 10 years (questionnaire)	Self-administration in-person	Daily sitting	1	Usual day, single day	Categorical (including: “almost all of the time, bout ¾ of the time, about ½ of the time, about ¼ of the time, almost none of the time”)
Canadian Census – Long Form	1996, 2001, 2006, 2016, ongoing	≥ 15 years	Self-administration in-person	Passive transit (usual transit mode to work)	1	Usual day	Categorical (including: car, truck or van - as driver; car, truck or van - as passenger; public transit; motorcycle, taxicab)
Canadian Community Health Survey	2000–01, 2003, 2005, 2007, 2008	≥ 12 years (excludes residents of Indian Reserves, Crown Lands, institutions, certain remote regions, full-time members of Canadian Forces, and beginning in 2015 excludes youth living in foster homes)	Interview-led, personal interviews that were computer-assisted and assessed by phone	1. Leisure computer 2. Leisure video games^a^ 3. Leisure television 4. Leisure reading (paper-based)	4	Typical week, past 3-months	Categorical (none, < 1 h/week, 1-2 h/week, 3-5 h/week, 6-10 h/week, 11-14 h/week, 15-20 h/week, > 20 h/week)
	2009, 2010			1. Leisure computer 2. Leisure video games^a^ 3. Leisure television 4. Leisure reading (paper-based)			Continuous
	2011, 2012, 2013, 2014			1. Leisure computer 2. Leisure video games^a^ 3. Leisure television 4. Leisure reading (paper + eBooks)			
	2015, 2016			1. Free time reading (including homework, paper-based and electronic formats) 2. Free time television, DVDs, movies or Internet videos 3. Free time video or computer games^a^ 4. Free time computer, tablet or smart phone		Last 7 days	Continuous
	2017, 2018, ongoing			1. Free time screen (television, electronic device) on school/work day 2. Free time screen (television, electronic device) on non-school/non-workday	2		Categorical (≤ 2 h/day, > 2 but < 4 h/day, 4 to < 6 h/day, 6 to < 8 h/day, ≥ 8 h/day)
Canadian Community Health Survey – Nutrition Focus Survey	2004	Children: 6–11 years Youth: 12–17 years	Interview-led, personal interviews that were computer-assisted and assessed by phone	Children: 1. Television, videos, video games 2. Computer (including playing games, e-mailing, chatting, surfing the Internet)	Children: 2	Children: average day	Children: categorical (none, <1 h/day, 1-2 h/day, 3-4 h/day, 5-6 h/day, ≥ 7 h/day)
				Youth: 1. Leisure computer 2. Leisure video games 3. Leisure television/videos 4. Leisure reading	Youth: 4	Youth: typical week, past 3-months	Youth: categorical (none, < 1 h/week, 1-2 h/week, 3-5 h/week, 6-10 h/week, 11-14 h/week, 15-20 h/week, > 20 h/week)
	2015	6–17 years		Total screen time (television, video games, computer, hand-held devices)	1	Average day, single day	Continuous (hours)
Canadian Community Health Survey – Aging	2008–2009	≥ 45 years	Interview-led, personal interviews that were computer-assisted and assessed by phone	Total sitting time (e.g., reading, watching television, computer activities or doing handicrafts) – based on Physical Activity Scale for the Elderly	3	Past 7 days	Categorical (including types of sitting and quantity)
				Occupational	1	Usual day, single day	Categorical (including “usually sitting”)
Canadian Health Measures Survey (CHMS)	2007–2009 (Cycle 1)	Children: 3-11 years (proxy) - note: for 2007-2009 Cycle 1, children includes ages 6-11 years only. Youth/adults: 12–79 years The survey excludes residents of Indian Reserves, the territories, institutions, certain remote regions, and full-time members of the Canadian Forces.	Interview-led, personal interviews that were computer-assisted and assessed by phone	Children: 1. Total sedentary time (accelerometer) 2. Total television or videos or video games 3. Total computer use Youth/adults: 1. Total sedentary time (accelerometer) 2. Leisure computer (incl. tablet) 3. Leisure video + console games 4. Leisure television (incl. DVDs, videos) 5. Leisure reading (paper-based)	Children: 2 Youth/adults: 4	Children: average day, single day Youth/adults: typical week, past 3-months	Children: categorical (0 h/day, < 1 h/day, 1–2 h/day, 3–4 h/day, 5–6 h/day, ≥ 7 h/day) Youth/adults: categorical (none, < 1 h/week, 1-2 h/week, 3-5 h/week, 6-10 h/week, 11-14 h/week, 15-20 h/week, > 20 h/week)
	2009–2011 (Cycle 2)			Children: 1. Total sedentary time (accelerometer) 2. Total television or videos or video games 3. Total computer use Youth/adults: 1. Total sedentary time (accelerometer) 2. Leisure computer (incl. tablet) 3. Leisure video + console games 4. Leisure television (incl. DVDs, videos) 5. Leisure reading (paper-based)			Children: categorical (0 h/day, < 1 h/day, 1–2 h/day, 3–4 h/day, 5–6 h/day, ≥ 7 h/day) Youth/adults: continuous
	2012–2013 (Cycle 3), 2014–2015 (Cycle 4)			Children: 1. Total sedentary time (accelerometer) 2. Total television or videos or video games 3. Total computer use Youth/adults: 1. Total sedentary time (accelerometer) 2. Total self-reported sitting time (IPAQ) in Cycle 3 only 3. Leisure computer (incl. tablet) 4. Leisure video + console games (Cycles 3 + 4 includes passive only) 5. Leisure television (incl. DVDs, videos) 6. Leisure reading (paper + eBooks, does not include reading on computer or Internet)			Children: categorical (0 h/day, < 1 h/day, 1 to < 3 h/day, 3 to < 5 h/day, 5 to < 7 h/day, ≥ 7 h/day) Youth/adults: continuous
	2016–2017 (Cycle 5), ongoing			Children: 1. Total sedentary time (accelerometer) 2. Total screen time (television/game console/computer/hand-held devices) Youth/adults: 1. Total sedentary time (accelerometer) 2. Free time computer (incl. tablet, smartphone) 3. Free time video + computer games 4. Free time television (incl. DVDs, movies, internet videos) 5. Free time reading (paper + electronic formats, excludes reading on a computer, tablet or Internet)	Children: 1 Youth/adults: 4	Children: average day, single day Youth/adults: past 7 days	Children: continuous Youth/adults: continuous
Canadian Health Survey on Children and Youth (CHSCY)	2016 (pilot), ongoing	1–17 years	Self-administration on-line	1. Homework (outside of class) 2. Reading 3. Social media use (Facebook, Snapchat, Instagram, Twitter, Pinterest) 4. Television (movies, videos, YouTube, television shows) 5. Video games 6. Any electronic device while sitting 7. Transportation mode (including passive)	20 (several multi-part questions)	Past 7 days, daily average	1–6: categorical, 7: continuous
Canadian Internet Use Survey (formerly Household Internet Use Survey)	1999	≥ 16 years	Electronic questionnaire or computer-assisted telephone interview	Internet use (frequency & amount)	2	Typical month	Categorical
	2000			Internet use (frequency & amount)	4		
	2001, 2002, 2003			Internet use (frequency & amount)	5		
	2005, 2007, 2009			Internet use (frequency & amount)	4	Typical month & typical week	
	2010, 2012			Internet use (frequency & amount)	3		
Canadian Longitudinal Study on Aging (CLSA)	2011 – ongoing	≥ 45 years	Interview-led, personal interviews that were computer-assisted and assessed by phone	Sedentary activities including: games, computer activities, crossword/puzzles, crafts, listening to music, playing musical instruments, reading, visiting with others, watching television, other	3	Average day in past 7 days	Categorical
General Social Survey – Time Use Survey (GSS)	1986, 1992, 1998, 2005, 2010, 2015, ongoing	≥ 15 years Living in private households in 10 provinces	Interview-led, personal interviews that were computer-assisted and assessed by phone	Activity recall over 24-h. Derived variables include: 1. Passive travel (car + bicycle + taxi + boat/ferry + airplane) 2. Watching television or videos 3. Reading (online or paper) *Note, the GSS also asks sedentary behaviour related questions on other years of the survey.*	N/A – diary	24-h period	Categorical selection of activity, continuous duration
Health Behaviour in School-Aged Children (HBSC) study	1990, 1994, 1998	Grades 6 to 10 (8 & 10 in 1994)	Self-administration in-person	1. Daily television watching 2. Weekly VCR movies 3. Weekly computer games (including arcade, game consoles)	3	Usual weekday and weekend day	1990, 1994, 1998: Television - categorical (none, < 0.5 h/day, 0.5-1 h/day, 2-3 h/day, 4 h/day, > 4 h/day) VCR movies and computer games - categorical (none, < 1 h/week, 1-3 h/week, 4-6 h/week, 7-9 h/week, ≥ 10 h/week) 2002, 2006, 2010, 2014: Categorical (none, ~0.5 h/day, ~1 h/day, ~2 h/day, ~3 h/day, ~4 h/day, ~5 h/day, ~6 h/day, ~7 or more h/day)
	2002	Grades 6 to 10		1. Free time daily television (including videos) 2. Daily homework 3. Free time daily computer use (playing games, emailing, chatting, surfing Internet)	6 (3 x weekday, 3 x weekend day)		
	2006, 2010			1. Free time daily television (including videos & DVDs) 2. Free time video games on computer or games console 3. Free time computer (chatting on-line, Internet, emailing, homework)	6 (3 x weekday, 3 x weekend day)		
	2014, ongoing			1. Free time daily television (including videos, DVDs, YouTube or other entertainment on screen) 2. Free time games on computer, games console, tablet, smartphone or other electronic device (not including active games) 3. Free time electronic device (computers, tablets, smartphones) use for other purposes (e.g., homework, emailing, tweeting, social media, chatting, surfing the internet)	6 (3 x weekday, 3 x weekend day)		
Health Promotion Survey	1985	≥ 15 years	Interview-led, personal interviews, computer-assisted and assessed by phone	1. Passive travel passenger (distance travelled) 2. Passive travel driver	2	Average	Continuous (distance travelled)
	1990			Daily activities	1	Usual	Categorical with *“You sit during the day and do not walk about very much”*
Joint Canada/United States Survey of Health (JCUSH)	2002–2003	≥ 8 years	Interview-led, personal interviews, computer-assisted and assessed by phone	Daily activities or work habits	1	Past 3 months	Categorical with *“Usually sit during the day and don’t walk around very much”*
National Longitudinal Survey of Children and Youth (NLSCY)	1994–1995, 1996–1997	Proxy: 0–9 years Self-reported: ≥ 10–11 year olds	Interviewer-administered in-person with computer-assisted interview	Proxy 1. Leisure computer or video games 2. Leisure television or videos 3. Leisure reading (by adult and child) 4. Homework Self-report 1. Leisure computer or video games 2. Leisure television 3. Leisure reading	Proxy: variable Self-report: variable	Variable	Proxy: mix of categorical and continuous Self-report: mix of categorical and continuous
	1998–1999	Proxy: 0–9 years Self-reported: ≥ 10–11 year olds		Proxy 1. Leisure television or videos 2. Leisure reading (by adult and child) 3. Homework 4. Writing Self-report 1. Leisure computer or video games 2. Leisure television 3. Leisure reading			
	2000–2001	Proxy: 0–9 years Self-reported: ≥ 10–11 year olds		Proxy 1. Leisure computer 2. Leisure reading (by adult and child) Self-report youth (age dependent) 1. Homework 2. Transportation mode 3. Computer or video games 4. Leisure writing 5. Leisure reading 6. Television and videos			
	2002–2003, 2004–2005	Proxy: 0–9 years Self-reported: ≥ 10–11 year olds		Proxy 1. Leisure computer 2. Leisure reading (by adult and child) 3. Leisure television or videos Self-report youth (age dependent) 1. Computer use 2. Internet use 3. Leisure writing 4. Leisure reading television, videos or video games			
	2006–2007, 2008–2009	Proxy: 0–9 years Self-reported: ≥ 10–11 year olds		Proxy 1. Leisure computer 2. Leisure reading (by adult and child) 3. Leisure television, videos or video games Self-report youth (age dependent) 1. Computer use 2. Internet use 3. Leisure writing 4. Leisure reading 5. Television, videos or video games			
National Population Health Survey (NPHS)	Cross-sectional & longitudinal: 1994–1995, 1996–1997, 1998–1999 Longitudinal only: 2000–2001, 2002–2003, 2004–2005, 2006–2007, 2008–2009, 2010–2011	All ages (only those ≥ 12 years asked sedentary questions)	Cycle 1: Interviewer-administered in-person with computer-assisted interviewing	1. Daily activities or work habits		Usual day over past 3 months	Categorical with “*Usually sit during day and do not walk about very much*” response option
			Cycle 2+: Interview-led, personal interviews, computer-assisted and assessed by phone				
National Household Survey (NHS)	2014	Children: 4–11 years Youth: 12–17 years	Self-administration in-person or on-line	1. Television, movies or videos (including YouTube) 2. Leisure computer, tablet, smartphone, video games	2	Past 7 days, average single day	Categorical
Survey of Young Canadians – Child	2010–2011	1–9 years	Interview-led, personal interviews, computer-assisted and assessed by phone	1. Leisure computer use 2. Television, videos or video games 3. Leisure reading	4	On average, single day	Categorical
Youth Smoking Survey	2002	Grades 5–9	Self-administration in-person	1. Computer or video games 2. Television or videos 3. Leisure reading	3	Computer and video games: last 12 months Television and reading: average day	Categorical

^a^Time spent playing video games was not consistently assessed in all age groups; from 2000 to 2001 to 2008 the questions were only asked to those under the age of 20, in 2009 and 2010 the questions were asked only to those under 25, whereas from 2011 to 2016 the questions were asked to all respondents

#### Canadian Community Health Survey (CCHS)

The CCHS began in 2000 and is an ongoing, cross-sectional survey conducted by Statistics Canada that collects health information from a representative sample of the Canadian private-dwelling population ages 12 and older. For some years, the module assessing sedentary activity was asked of all respondents and for other years it was optional content selected by a subset of health regions or provinces and is only representative of those regions (Additional file 1: Table S1).

#### Canadian Health Measures Survey (CHMS)

The CHMS began in 2007 and collects self-reported and measured health information from a representative sample of the Canadian household-dwelling population aged 3 (6 to 79 years in Cycle 1 to 79 years). The CHMS collects data from an interview-administered questionnaire conducted in the respondent’s home, as well as from a visit to a mobile examination centre (MEC) where physical measures are taken. The parent/guardian of children aged 3–11 years answer the household questionnaire on their behalf. During their visit to the MEC, ambulatory respondents of the CHMS were invited to wear an Actical accelerometer (Philips Respironics, Oregon, United States) over their right hip during waking hours for seven consecutive days. The Actical records time-stamped acceleration in all directions and provides an index of movement intensity based on a count-per-minute value; an intensity of < 100 counts-per-minute was used to identify sedentary time [23, 24]. A complete description of accelerometer data reduction procedures is available elsewhere [23–25].

#### General Social Survey (GSS) – Time-Use Surveys

The GSS collects information on living conditions, social life, well-being of Canadians, and specific social policy issues. Time-Use surveys began in 1985 and are conducted at approximately five- to seven- year intervals and employ a retrospective 24-h time diary to collect information on respondents’ participation in, and time spent on, a wide variety of day-to-day activities. Starting in 2010, it accounted for multitasking. This analysis includes data from the cycles of the GSS with a time-use survey. Within the GSS, ‘youth’ only includes those aged 15 to 17 years.

#### Health Behaviour in School-Aged Children Survey (HBSC)

The HBSC is a nationally representative survey that collects information on the health and well-being, social environments and health behaviours of school students [26–28]. The HBSC is a collaborative study with the World Health Organization Regional Office for Europe with 49 participating countries across Europe and North America and collects data every four years on 11-, 13- and 15-year old boys and girls. The PHAC funds the Canadian component of the HBSC which is conducted by Queen’s University and began in 1994.

### Statistical analyses

All analyses were conducted using SAS Enterprise Guide v.5.1 (SAS, Inc., Cary, NC). Descriptive statistics summarized amounts of various total sedentary time and specific sedentary activities (e.g., passive travel, leisure television, screen, computer, video games, and reading), across surveys using means or proportions and 95% confidence intervals (CIs). Cases missing data per sedentary behaviour outcome were omitted from the respective analyses. Using unadjusted linear regression analyses crude trends in average daily accelerometer-measured sedentary time across cycles were examined within each age group. Between-cycle pairwise contrasts were conducted to examine differences between cycles with a Bonferroni adjustment. Within all surveys, if a categorical response option with a range was included, we used the midpoint of each category to generate a continuous measure of time spent in the various sedentary activities (e.g., 0–1 h = 0.5 h), for the uppermost category we used the starting amount (e.g., more than 20 h = 20 h). Time spent in various screen-based activities was summed to provide a measure of total leisure screen time per day.

Where possible, the number of children and youth meeting screen time recommendations (≤ 2 h/day of recreational screen time) from the Canadian 24 Hour Movement Guidelines for Children and Youth [29] were estimated using proportions and 95% CIs. Results are presented by age group with between group differences assessed using independent sample t-tests or analysis of variance for continuous outcomes or chi-square for categorical outcomes. In the figures presented, data points that are derived from the same questions and response options have been connected with a solid line.

To account for the complex survey design and non-response bias and to correctly estimate variance, all analyses of CCHS [30] and CHMS [31] data were weighted using the survey weights generated by Statistics Canada. For the CHMS data, denominator degrees of freedom were set at 11 for all cycles except for Cycle 2 which used 13. To account for survey design effects in the CCHS, CHMS and GSS, the bootstrap technique was used to estimate 95% CIs. In the first five cycles the HBSC generated an unweighted national sample. Analysis of data from 2010 onwards incorporates population weights and controls for clustering at the school level.

## Results

### Overview of surveys

**Fig. 1:**
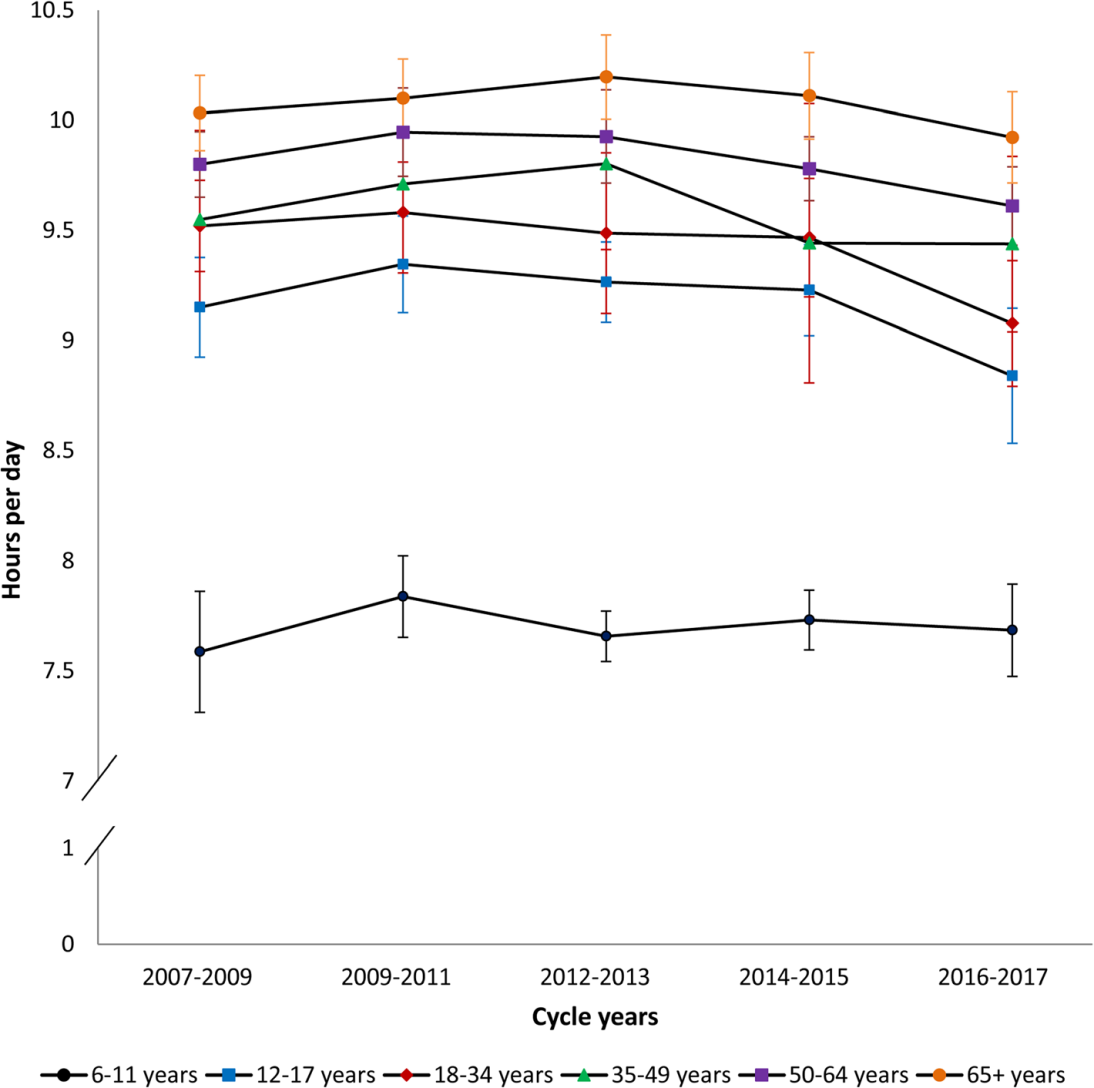
Accelerometer-measured total sedentary time by age group. Data sources: Canadian Health Measures Survey (2007–2009, 2009–2011, 2012–2013, 2014–2015, 2016–2017)

**Fig. 2:**
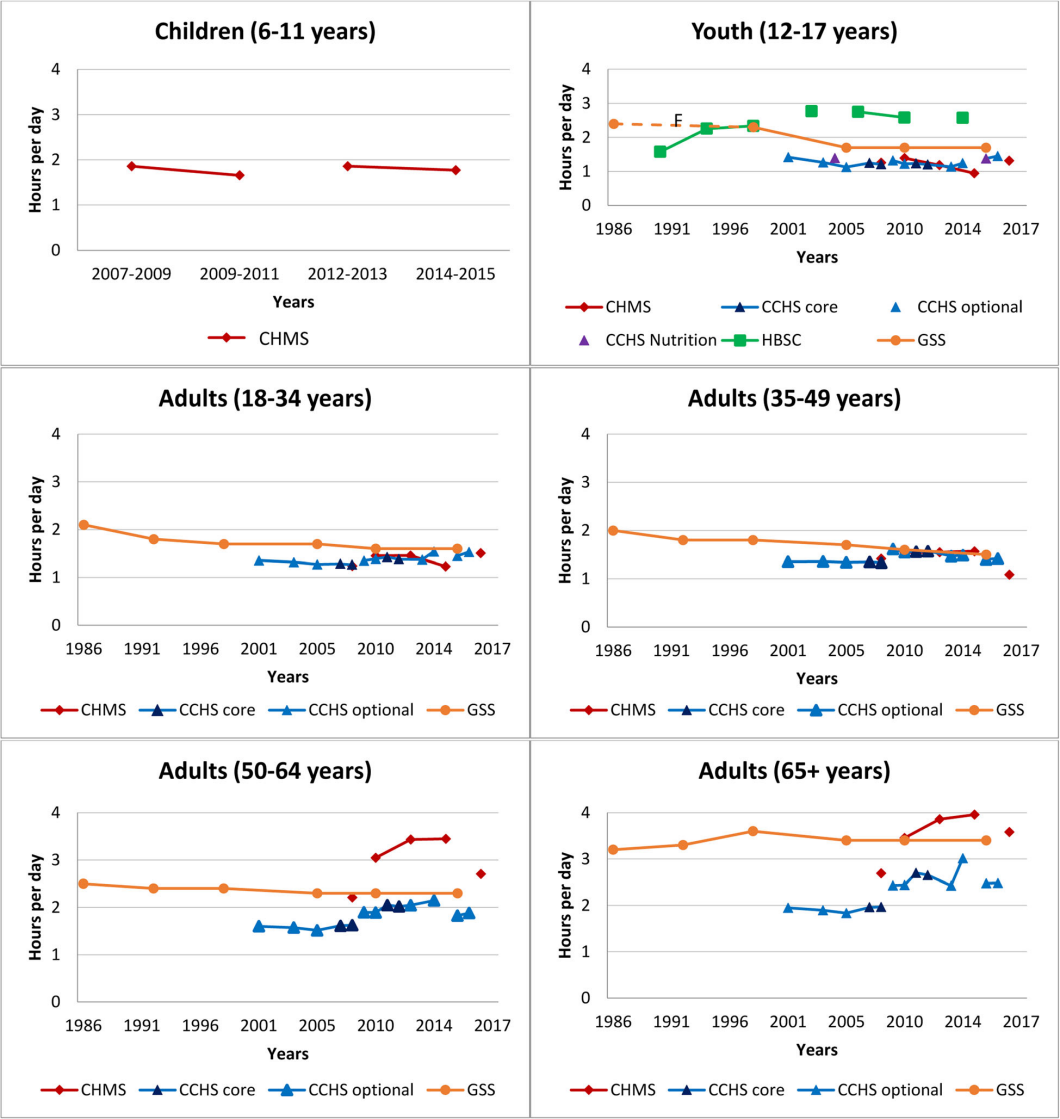
Self-reported daily average leisure television or video watching time by age group across survey years. Legend: F – Estimate not releasable. Data sources: Canadian Community Health Survey Annual – Core content (CCHS core; 2007–2008, 2011–2012); Canadian Community Health Survey Annual – Optional content (CCHS optional; 2000–01, 2003, 2005, 2009, 2010, 2013, 2014, 2015, 2016); Canadian Community Health Survey – Nutrition Focus Survey (CCHS Nutrition; 2004, 2015); Canadian Health Measures Survey (CHMS; 2007–2009, 2009–2011, 2012–2013, 2014–2015, 2016–2017); General Social Survey – Time Use Survey (GSS; 1986, 1992, 1998, 2005, 2010, 2015), Health Behaviour in School-Aged Children study (HBSC; 1990, 1994, 1998, 2002, 2006, 2010, 2014). Only surveys with the same questions and responses have been joined by solid lines

**Fig. 3:**
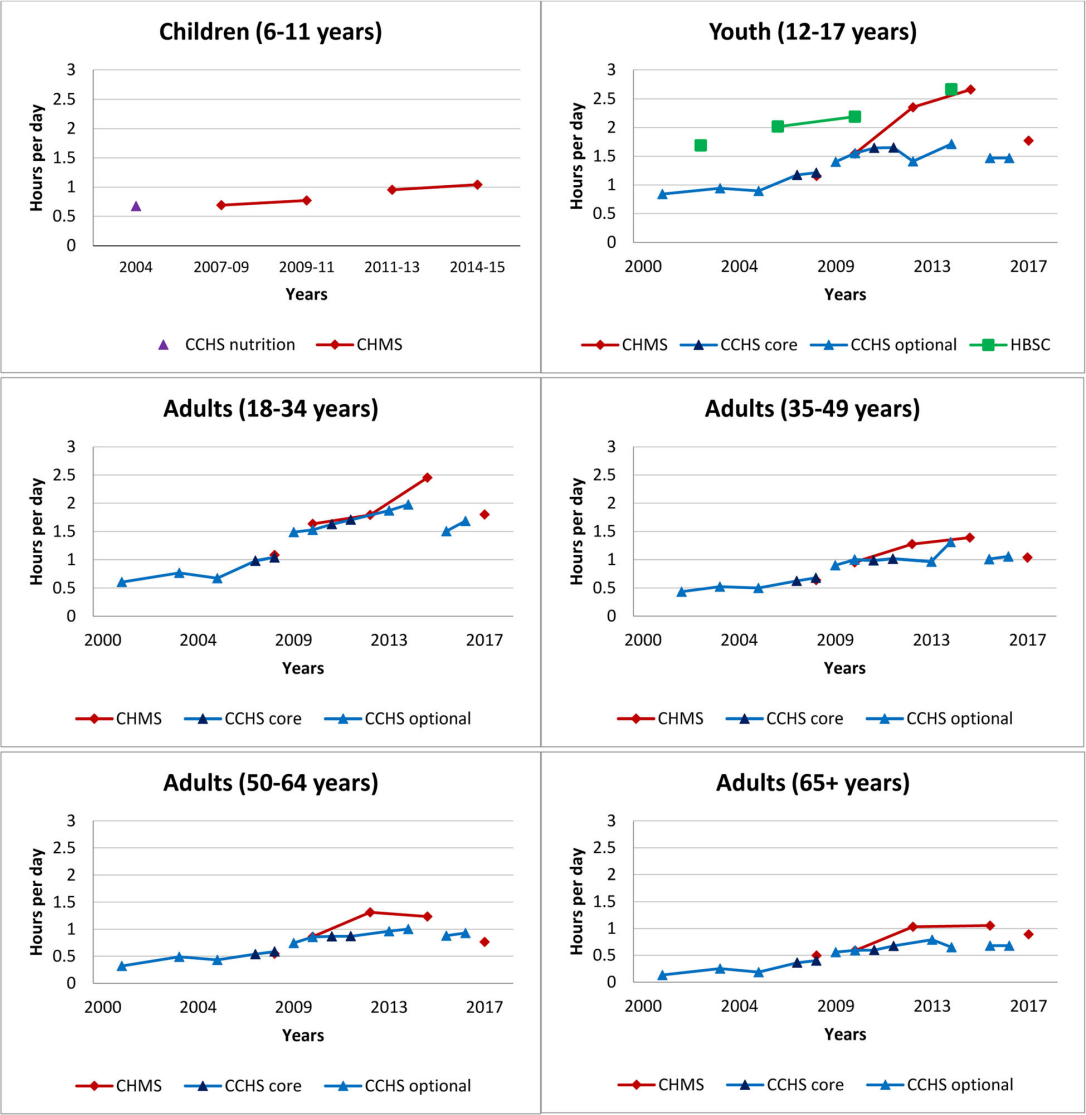
Self-reported daily average leisure computer time by age group across survey years. Data sources: Canadian Health Measures Survey (CHMS; 2007–2009, 2009–2011, 2012–2013, 2014–2015, 2016–2017); Canadian Community Health Survey Annual – Core content (CCHS core; 2007–2008, 2011–2012); Canadian Community Health Survey Annual – Optional content (CCHS optional; 2000–01, 2003, 2005, 2009, 2010, 2013, 2014, 2015, 2016); Canadian Community Health Survey – Nutrition Focus Survey (CCHS Nutrition; 2004 and 2015); Health Behaviour in School-Aged Children study (HBSC; 1990, 1994, 1998, 2002, 2006, 2010, 2014). Only surveys with the same questions and responses have been joined by solid lines

**Fig. 4:**
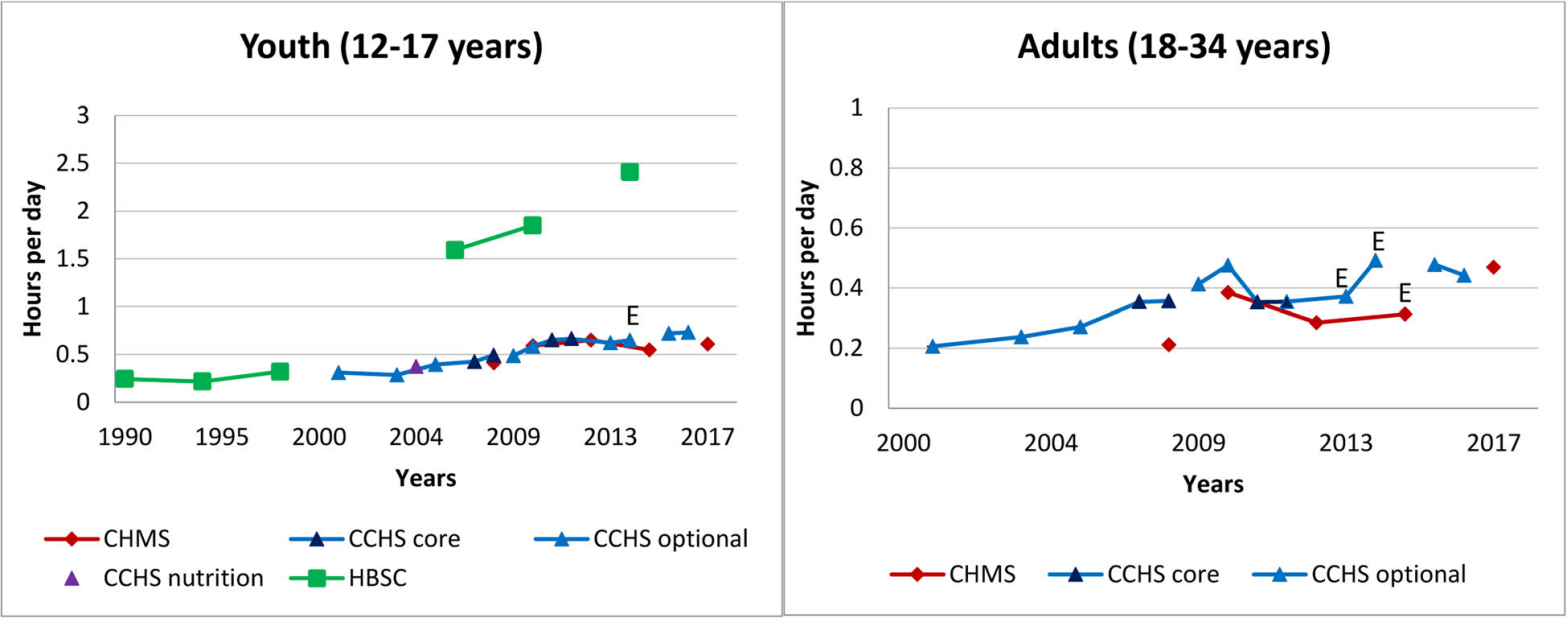
Self-reported daily average leisure video game time in youth and young adults across survey years. Legend: E – Interpret estimate with caution due to high sampling variability. Data sources: Canadian Community Health Survey Annual – Core content (CCHS core; 2007–2008, 2011–2012); Canadian Community Health Survey Annual – Optional content (CCHS optional; 2000–01, 2003, 2005, 2009, 2010, 2013, 2014, 2015, 2016); Canadian Community Health Survey – Nutrition Focus Survey (CCHS Nutrition; 2004, 2015); Canadian Health Measures Survey (CHMS; 2007–2009, 2009–2011, 2012–2013, 2014–2015, 2016–2017); Health Behaviour in School-Aged Children study (HBSC; 1990, 1994, 1998, 2002, 2006, 2010, 2014). Only surveys with the same questions and responses have been joined by solid lines

**Fig. 5:**
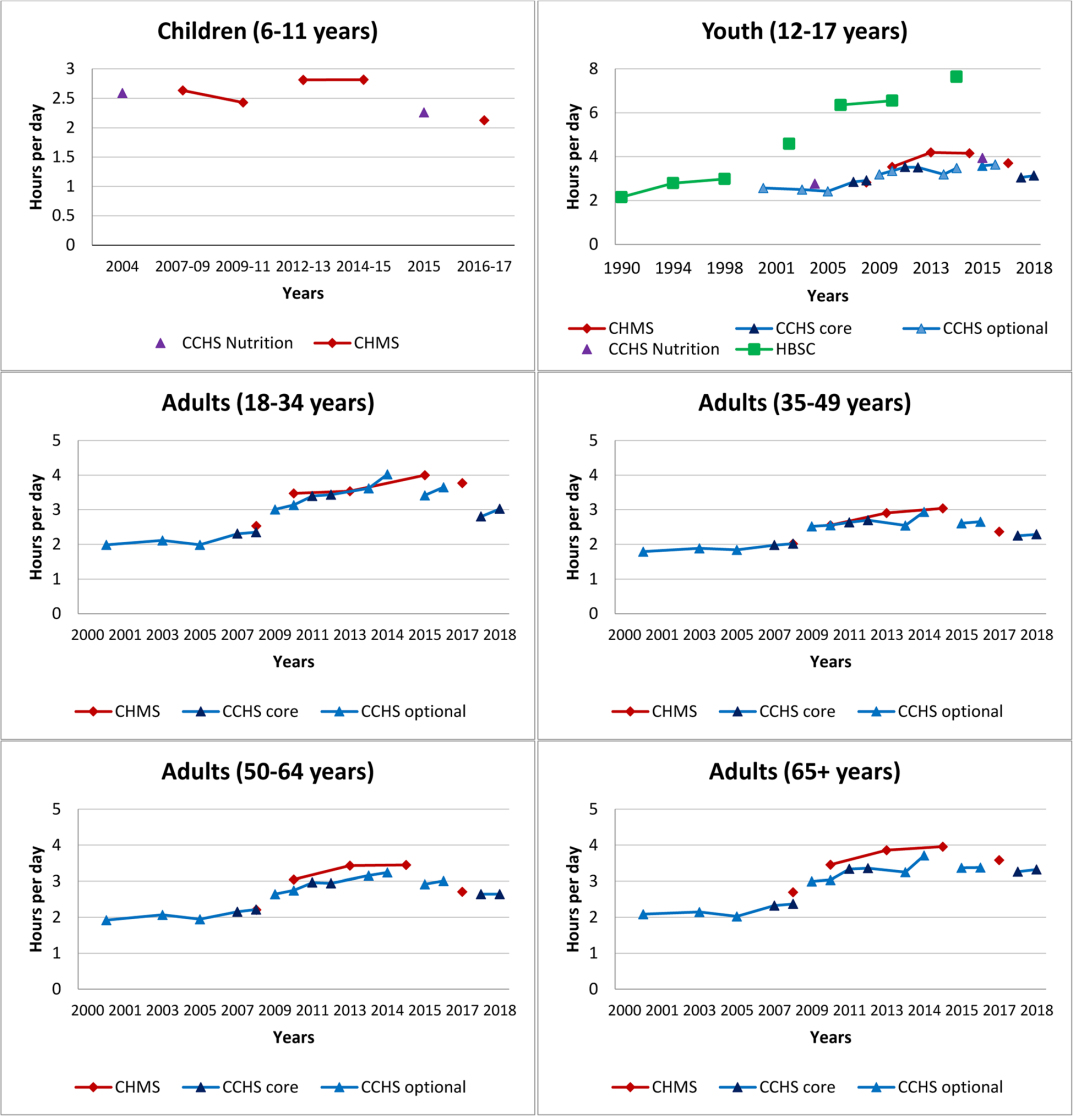
Self-reported daily average leisure screen time by age group across survey years. Data sources: Canadian Health Measures Survey (CHMS; 2007–2009, 2009–2011, 2012–2013, 2014–2015, 2016–2017); Canadian Community Health Survey Annual – Core content (CCHS core; 2007–2008, 2011–2012, 2017–2018); Canadian Community Health Survey Annual – Optional content (CCHS optional; 2000–01, 2003, 2005, 2009, 2010, 2013, 2014, 2015, 2016); Canadian Community Health Survey – Nutrition Focus Survey (CCHS Nutrition; 2004, 2015); Health Behaviour in School-Aged Children study (HBSC; 1990, 1994, 1998, 2002, 2006, 2010, 2014). Only surveys with the same questions and responses have been joined by solid lines

**Fig. 6:**
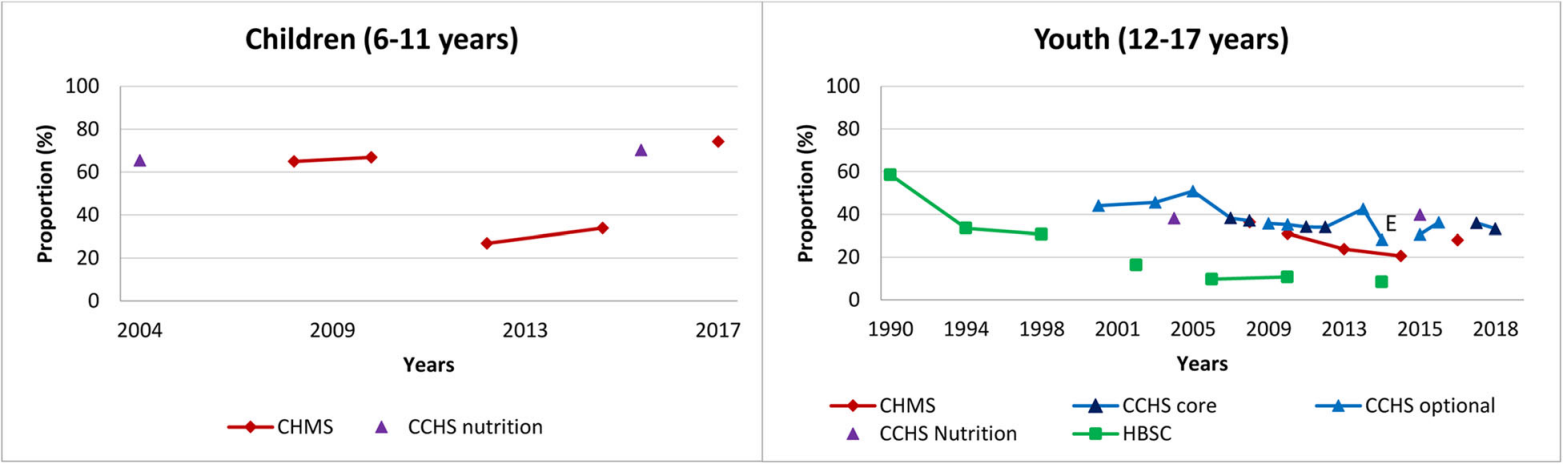
Proportion meeting screen time guideline adherence (≤ 2 h/day) by age group across survey years. Legend: E – Interpret estimate with caution due to high sampling variability. Data sources: Canadian Community Health Survey Annual – Core content (CCHS core; 2007–2008, 2011–2012, 2017–2018); Canadian Community Health Survey Annual – Optional content (CCHS optional; 2000–01, 2003, 2005, 2009, 2010, 2013, 2014, 2015, 2016); Canadian Community Health Survey – Nutrition Focus Survey (CCHS Nutrition; 2004, 2015); Canadian Health Measures Survey (CHMS; 2007–2009, 2009–2011, 2012–2013, 2014–2015, 2016–2017); Health Behaviour in School-Aged Children study (HBSC; 1990, 1994, 1998, 2002, 2006, 2010, 2014). Only surveys with the same questions and responses have been joined by solid lines

**Fig. 7:**
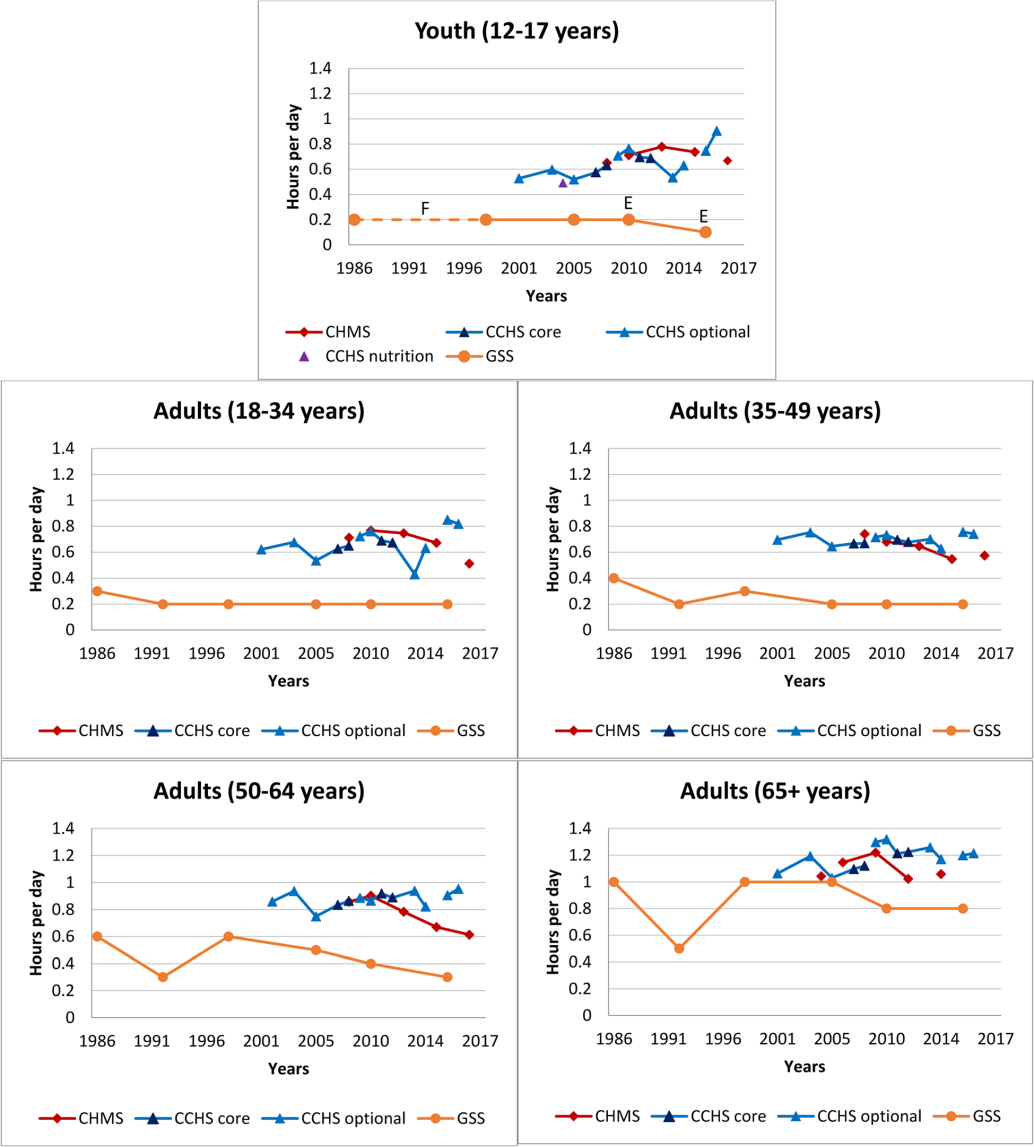
Self-reported daily average leisure reading time by age group across survey years. Legend: E – Interpret estimate with caution due to high sampling variability. F – Estimate not releasable. Data sources: Canadian Community Health Survey Annual – Core content (CCHS core; 2007–2008, 2011–2012); Canadian Community Health Survey Annual – Optional content (CCHS optional; 2000–01, 2003, 2005, 2009, 2010, 2013, 2014, 2015, 2016); Canadian Community Health Survey – Nutrition Focus Survey (CCHS Nutrition; 2004); Canadian Health Measures Survey (CHMS; 2007–2009, 2009–2011, 2012–2013, 2014–2015, 2016–2017); General Social Survey – Time Use Survey (GSS; 1986, 1992, 1998, 2005, 2010, 2015). Only surveys with the same questions and responses have been joined by solid lines

**Fig. 8:**
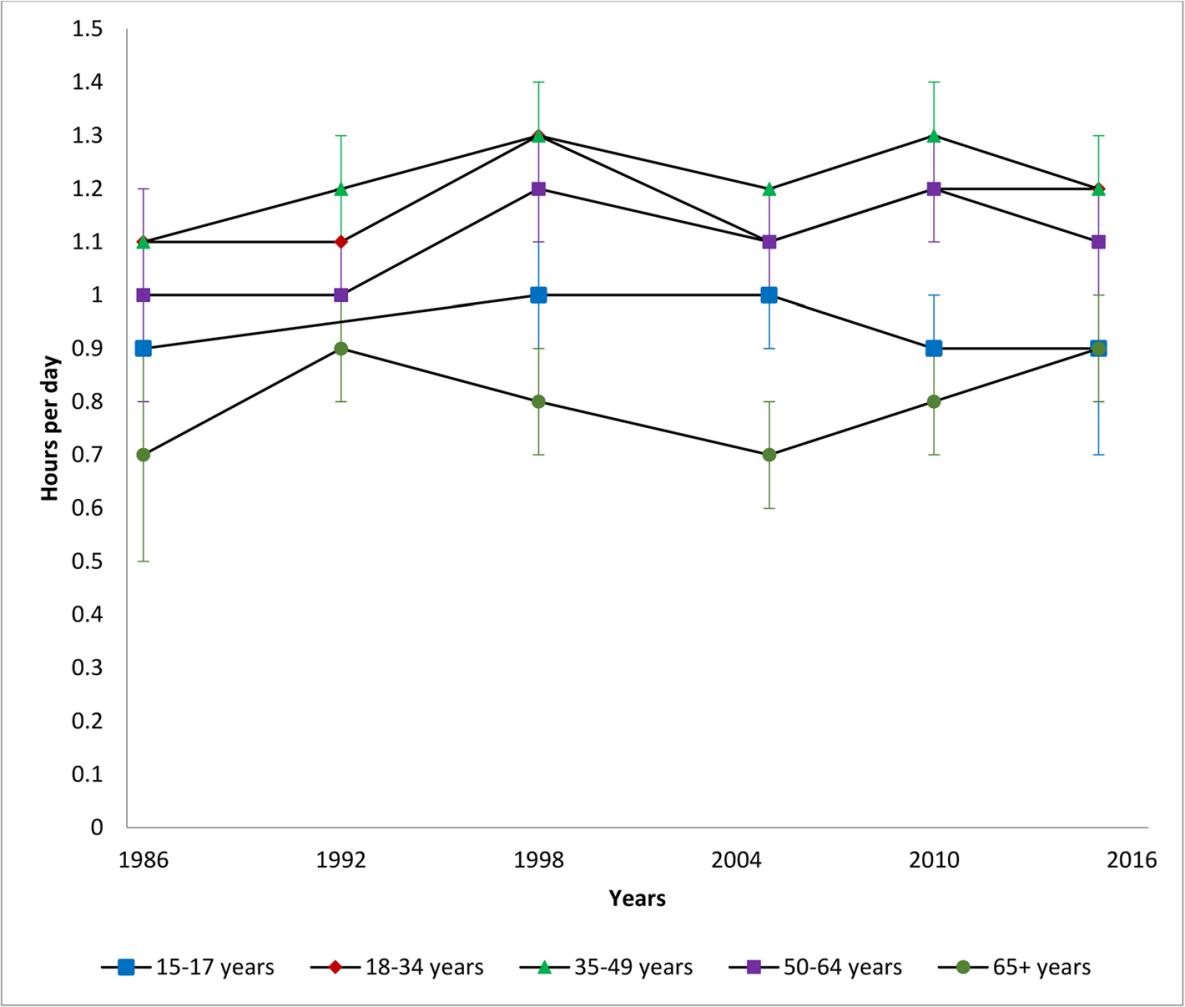
Self-reported daily average passive travel time by age group in the General Social Survey – Time Use Survey. Data sources: General Social Survey – Time Use Survey (1986, 1992, 1998, 2005, 2010, 2015)

Table 1 provides characteristics using the TASST framework for the 19 identified surveys. Canada’s long-standing history of collecting data on sedentary behaviour has lacked consistency across and within surveys, and several only administered one-to-two times. Although most surveys incorporate(d) a repeated cross-sectional design providing estimates over time in the Canadian population (or a sub-sample), changes in questions and/or response categories make it difficult to directly assess trends (see Table 1). Nonetheless, we present cross-sectional estimates by survey year for specific sedentary activities to provide a visual pattern of changes over time using data from the CCHS, CHMS, GSS, and HBSC (Figs. 1, 2, 3, 4, 5, 6, 7 and 8).

### Accelerometer-measured total sedentary time

Figure 1 presents total accelerometer-measured sedentary time by age groups across five cycles of the CHMS from 2007 to 2017. A significant negative linear trend for daily sedentary time in youth (β = − 0.08, *p* = 0.04), adults aged 18–34 years (β = − 0.10, *p* = 0.01), and adults aged 50–64 years (β = − 0.06, *p* = 0.03) was observed with an average decline of approximately 5 min/day per cycle. Pairwise contrasts, however, identified no significant between cycle differences. Sedentary time is higher in older age groups, with children engaging in significantly lower amounts and older adults engaging in significantly higher amounts compared to other age groups. In Cycle 5, sedentary time ranged from 7.7 (95% CI: 7.5–7.9) hours/day in children (6–11 years) to 9.9 (95% CI: 9.7–10.1) hours/day in older adults.

### Screen-based sedentary behaviours

Screen time questions or response options have changed frequently within surveys; resulting in discontinuity in trends and making it difficult to ascertain whether differences observed over time are a result of actual changes in the behavioural patterns or a result of variations in survey methodology (see Table 1 for changes in questions).

#### Watching television or videos

Figure 2 provides a historical overview of leisure time spent watching television and/or videos by age group across surveys. Most recent estimates of daily television and video watching time ranged from 1.3–1.7 h/day in youth, 1.5–1.6 h/day in adults 18–34 years, 1.1–1.5 h/day in adults 35–49 years, 1.8–2.7 h/day in adults 50–64 years, and 2.5–3.6 h/day in adults ≥ 65 years, depending on the survey. Television and video watching is highest in older adults and has increased over time, whereas it has declined or remained relatively stable across younger age groups.

#### Computer use outside of school or work

Figure 3 provides a historical overview of leisure time spent using a computer by age groups across surveys. Leisure computer use appears to have risen over time. Most recent estimates of daily leisure computer use ranged from 1.5–2.7 h/day in youth, 1.7–1.8 h/day in adults 18–34 years, 1.0–1.1 h/day in adults 35–49 years, 0.8–0.9 h/day in adults 50–64 years, and 0.7–0.9 h/day in adults ≥ 65 years, depending on the survey. Leisure computer use is consistently highest in youth (12–17 years) and young adults (18–34 years) and decreases with age. Among youth, reported estimates in the HBSC were considerably higher than those observed in the CCHS and CHMS.

#### Video game play

Figure 4 provides a historical overview of leisure time spent playing video games in youth and young adults (18–34 years) across survey years. We have omitted those aged ≥ 35 years due to low prevalence and high variability in the estimates of video game play. Although the surveys collected data on both passive (i.e., done while sitting) and active (i.e., require physical activity) video games; here we focus on passive video games. Within the CHMS, parents were asked about children’s screen use including television or videos or video games, limiting the separate reporting of video game use in this population. Video game play is the least prevalent of sedentary activities, but appears to have risen over time. In the 2016–2017 CHMS, video game play was highest in youth (0.6 h/day, 95% CI: 0.5–0.8) and lowest in adults 50–64 years (0.2 h/day, 95% CI: 0.1–0.2). In youth, estimates are much higher in the HBSC survey from 2006 onwards compared to the CCHS and CHMS. Earlier cycles of the HBSC asked respondents about computer game use, whereas later surveys asked about combined video and computer game use and in 2014 also asked about games played on other electronic devices such as tablets and smartphones. In youth, the most recent estimate from the 2014 HBSC was 2.4 (95% CI: 2.3–2.5) hours/day – much higher than the corresponding 2014–2015 CHMS estimate of 0.6 h/day (95% CI: 0.4–0.7).

#### Total leisure screen time

Figure 5 provides a historical overview of leisure screen time by age group across survey years. Total leisure screen time was identified using a sum of time spent on different screen-based media depending on survey and year (see Table 1). A transition towards asking respondents to estimate their total leisure screen time rather than separate activities occurred in both the CCHS (starting in 2017) and CHMS (2016–2017 children). Despite the changes in survey questions, trends in self-reported daily leisure screen time appear to be increasing in all surveys.

Within each survey total screen time assessed via similar questions appears to have increased across all age groups. Among youth, daily leisure screen time from the HBSC has consistently been higher than that reported in the CCHS and CHMS. Most recent estimates of total daily leisure screen time ranged from 2.1–2.3 h/day in children, 3.1–7.6 h/day in youth, 2.8–3.8 h/day in adults 18–34 years, 2.3–2.4 h/day in adults 35–49 years, 2.6–2.7 h/day in adults 50–64 years, and 3.3–3.6 h/day in adults ≥ 65 years, depending on the survey. Youth appear to be the greatest leisure users of screens.

Figure 6 provides an overview of the proportion of children and youth meeting the screen time recommendations (≤ 2 h/day) from the Canadian 24-Hour Movement Guidelines [9]. The change in methodology has created a challenge in discerning whether the proportion of children and youth meeting the screen time guidelines has changed with time. Prior to more recent years (which reflect substantial changes to questions), adherence to screen time recommendations appeared to be declining.

### Reading time

Figure 7 provides a historical overview of leisure reading time by age groups across surveys. Reading outside of school or work is less prevalent than screen use. Reading in the CCHS and CHMS was largely paper-based with the introduction of eBooks in 2011 and 2012, respectively (note both surveys excluded time spent reading on the computer and Internet in later years). The GSS provides the most consistent assessment of reading time from 24-h time-use recalls, but included on-line reading. In youth, and younger adults (18–49 years), reading appears to have remained fairly constant or with minimal declines. Declines of approximately 18 and 12 min/day were observed between 1986 and 2015 in those aged 50–64 and ≥ 65 years, respectively. Within the most recent GSS (2015), levels of reading increase with age; 15–17 years (0.1 h/day, 95% CI: 0.1–0.2), 18–34 years (0.2 h/day, 95% CI: 0.1–0.2) and 35–49 years (0.2 h/day, 95% CI: 0.1–0.2), 50–64 years (0.3 h/day, 95% CI: 0.3–0.3), and ≥ 65 years (0.8 h/day, 95% CI: 0.7–0.8). The GSS often yielded significantly lower levels of reading than those reported in the CCHS and CHMS.

### Passive travel time

Figure 8 provides an overview of passive travel time by age groups over time in the GSS. Time spent in passive travel appears to have inclined slowly over the past three decades with variability between years. Older adults report the lowest amounts of passive travel; significantly lower than those aged 18–49 years across all years. Adults aged 35–49 years have the highest levels of passive travel over time; significantly higher than youth and older adults across all years. In 2015, passive travel estimates ranged from a low of 0.9 h/day (95% CI: 0.8–0.9) in older adults to 1.2 h/day in adults aged 18–34 years (95% CI: 1.1–1.2) and 35–49 years (95% CI: 1.2–1.3).

## Discussion

This is the first comprehensive review of sedentary behaviour surveillance data in Canada. Surveys differ with respect to their measures (e.g., questionnaire, 24-h recall, activity monitors), sampling frame (e.g., national level [CHMS], sub-national [CCHS]), and sample age (e.g. HBSC: youth in grades 6–10, CCHS: ≥ 12 years, CHMS: 3–79 years and GSS: ≥ 15 years). While this paper is focused on Canadians, the findings and lessons learned regarding measurement and surveillance are relevant to other countries with similar measures and surveys. We hope that the findings will help to inform interpretation of data in the surveillance of sedentary behaviour.

Assessing trends in type-specific sedentary activities (i.e., television/video, computer, video games, reading) is difficult given the changing nature of the content and this precluded our ability to report with certainty on changes in estimates over time. At times, different data sources told different stories, adding to the complexity of interpretation. For example, in Fig. 2 we see that among adults aged 50–64 and ≥ 65 years, television viewing increased in the CCHS and CHMS, but remained relatively stable in the GSS. Research in older adults using the TASST framework found that using a recall period of a typical day in the past week was more sensitive to change than a past day recall [32].

Similar to what was observed in the United States using data from the National Health and Nutrition Examination Survey (2001–2016); television time appears to have remained relatively stable (amongst most age groups), while computer and electronic device use has increased [19]. Also similar to what was observed in the Australian Time Use Surveys; total leisure screen time looks to have increased [15]. A larger proportion of leisure time is spent engaged with different types of screens (e.g., tablets, smartphones) for different purposes (e.g., online games, shopping, communication). A temporal analysis of GSS data found that the proportion of Canadians reporting the use of computers during free time (e.g. email, on-line social networking, searching for information) increased nearly five-fold from 5% in 1998 to 24% in 2010 [33]. This uptake in screen time has implications given its association with chronic disease and mortality [3].

Leisure reading remained relatively stable in those younger than 50 years and although it appears to have declined in those older, their levels still remain higher. In comparison, the Australian Time Use Survey data suggest small reductions [15]. Reading is the only sedentary activity shown to be associated with beneficial outcomes including academic achievement and maintenance of cognitive function [34, 35].

Pairwise contrasts between cycles found no significant differences in total sedentary time. While there were significant negative linear trends in youth, young adults and adults aged 50–64 years, the only cycle to exhibit a lower estimate was the most recent cycle (2016–2017). Longer and subsequent follow-up would be needed to identify if a trend truly exists or if this is an artefact. We are unaware of any other national or international studies to have examined trends in device-measured sedentary time. However, others have examined total self-reported sitting; and have found that it has remained fairly stable [19, 36].

Within the GSS, passive travel time appears to have increased slowly. Other surveillance studies have also found that passive travel increased, albeit at a slower rate than screen use [15, 16]. The estimates produced reflect all passive travel and not solely commute time to work/school. According to data from the 2016 Census, Canadians (≥ 15 years) commuters spent an average of 26 min travelling to their work. From 2011 to 2016, the number of Canadians with longer commutes (≥ 60 min) rose by 5% [37, 38].

Historically, epidemiologic studies have largely relied on self-report measures of sedentary behaviour. Self-report measures are prone to systematic errors due to a respondent’s inability to accurately estimate their frequency and duration of time spent in behaviours [39]. Recall frame relates to the number of hours, days or weeks an individual recalls a behaviour in the past. While longer recall frames are used to provide a better estimate of ‘usual’ activity, shorter recall frames are used to improve reliability and validity of a questionnaire. Earlier cycles of the CCHS and CHMS asked participants to recall their sedentary activities during *a usual week over the previous 3 months*. Asking individuals to provide a summative estimate of weekly sedentary activities (e.g., television watching) is likely to increase information bias [39]. Recent modifications to reduce this recall frame to a *typical day in the past 7 days* have likely led to improvements in recall accuracy. It is difficult to establish the construct validity of self-report measures of domain- and type-specific sedentary activities as this would require them to be compared to an accurate measure of the behaviour (e.g., combination of GPS and accelerometer, body camera, direct observation). However, criterion or predictive validity has been assessed demonstrating that different doses of these type-specific sedentary activities (e.g., screen time) are associated with health outcomes [35, 40–43].

The 24-h activity recall data collected within the GSS Time Use Survey is likely to provide a more accurate estimation of time spent in various behaviours versus questionnaire. Given that 24-h is a finite amount of time, respondents must consider their entire day and allocate activities accordingly. Time-use diaries have been shown to be more valid than questionnaires at quantifying time spent sedentary [44, 45]. Unfortunately, time-use surveys are resource intensive placing a greater burden upon respondents, as well as those who undertake analysis. Given that space on a population health survey is always limited, the capacity to undertake these recalls is unlikely [46]. Balancing the needs and interests of multiple data users (e.g., surveillance, policy makers and researchers) in sedentary behaviour surveillance is a challenge. In our previous work [46], we describe that sedentary behaviour questions should be “concise, valid/reliable, evidence-based, and developed using best practices”. Questions should also be “adaptable and able to assess various modes of sedentary behaviour” [14]. The International Sedentary Assessment Tool (ISAT) is a sedentary behaviour module that addresses these needs by providing: an itemized list of questions in order of their established relationship with health; the capacity to use any item separately; and, examples of modes (e.g., Smartphone, tablet) in brackets to allow for updates as new technologies/modes emerge [14]. Additionally, it recommends collecting responses in continuous format to allow for guideline assessment in the future [14].

Only recently have surveillance systems included the use of device-measured (e.g., accelerometer) sedentary time to overcome some of the potential sources of bias in self-report measures [39]. The CHMS strengthens Canadian surveillance by monitoring total sedentary time and patterns of behaviour (e.g., breaks, bouts) using accelerometers. While accelerometers help to reduce response bias, they may misclassify time spent standing stationary as sedentary time. It is important that as the field moves to also include wrist-worn accelerometers (which have shown to improve compliance in wearing the monitor) [47] that surveillance systems consider the accuracy of these devices for detecting sedentary time in addition to physical activity and how this will affect trends. While this is a rich data source that can provide more accurate information on the sedentary time of Canadians, the CHMS is not able to provide sub-national estimates (e.g., provincial/territorial) and lacks the ability to distinguish between domains and types of sedentary activities. This domain- and type-specific information is important for designing interventions and identifying key targets for behavioural modification. The complimentary nature of the self-reported and device-measured data can offer up rich insights into the behaviours of Canadians and other countries.

Ensuring that methodologies used to assess sedentary behaviour are consistent enhances the ability of a surveillance system to examine the behaviour in relation to health outcomes and assess differences across demographics. Surveillance systems benefit from questions that are sensitive to change, provide reliable estimates and sufficient face (i.e. are the questions understood by respondents) and predictive validity (i.e. can identify a dose-response between behaviour and outcome). Predictive validity is one of the key components for establishing causality as outlined by the Bradford Hill criteria [48]. Self-reported sedentary behaviour is often more strongly associated with health outcomes than device measures [35, 49–51]. Recall of a specific sedentary activity, such as television, is likely easier than a summation of all time spent sitting throughout the day, and has been found to be more strongly associated with health outcomes than total sitting [6]. Evidence suggests that a single question for total sitting is less valid than a summative measure when compared to a construct standard (e.g., activPAL or accelerometer) [52–55]. Recently the CCHS and CHMS have moved toward a single question for leisure screen time. This helps to overcome issues of multi-tasking where an individual may inadvertently ‘double-count’ time spent on multiple devices such as watching television while simultaneously using a tablet to surf the Internet. It may explain why estimates of total leisure screen time in the newer cycles are lower than those preceding where a summation of television, computer and video game time was used. It may also be that asking individuals to recall all of their leisure screen time increases the likelihood of response and recall bias compared to asking about activities separately.

### Strengths and limitations

Strengths of this study include the use of data from large, population-based survey samples and examination of trends by age group. The study examined trends in device-measured total sedentary time which had not previously been done in a national cohort and which helped to overcome biases associated with self-reported total sitting. Previous examinations of trends in sitting time in other international cohorts have relied on self-reported measures [19, 33]. This study also examined trends for a number of self-reported sedentary activities including ‘inactive’ video games which have not yet been examined in a national cohort. Unfortunately, we were unable to report on trends in occupational sedentary time. To date, no known Canadian national survey has collected information on time spent sitting exclusively at school or work. In the years covered by this analysis, the technologies used for screen time have proliferated making older measurement methodologies less able to reflect current technology use. These changes largely prevented the assessment of trends using statistical comparisons given that measures have changed over time. Our results focus on Canadians aged ≥ 6 years. There is very little national data for sedentary behaviour among infants and preschool-aged children and even among those aged 6–11 years there are few sources of data for some indicators (e.g., television and video watching). This is a limitation of the current surveillance landscape both in Canada and abroad. We hope that the new Canadian Health Survey on Children and Youth [56] will help to fill in some of these gaps.

## Conclusions

We hope this inventory of measures demonstrates opportunities and challenges of measuring an evolving concept like sedentary behaviour through surveys. Although it seems like having static questions would be helpful for monitoring trends over time, it is not appropriate to ignore changes in behaviours and preferences. International surveillance systems can apply the lessons learned within this paper when examining trends and comparing internally and to other countries. While total device-assessed sedentary time has remained relatively stable, time spent in self-reported leisure sedentary activities appears to have changed. Youth are the greatest leisure screen users and appear to be changing the types of screens with which they engage; swapping television viewing for greater time spent on computers and other electronic devices. While older adults continue to have the lowest amounts of leisure screen time, they appear to be decreasing their time spent reading and increasing their time spent watching television and using electronic devices, which based on the evidence is a significant public health concern. Total leisure screen use appears to have increased over time a reflection of the changing ways in which Canadians spend their free time and technological advances. Future research into the risks associated with various types of sedentary activities and prolonged sedentary time and the health of Canadians (and other nations) across all ages (especially younger children) is needed.

## Supplementary information

**Additional file 1: Table S1.** Sedentary activities module uptake by region in the Canadian Community Health Survey.

## Data Availability

The datasets analysed during the current study are available through the Research Data Centres (RDC) Program at Statistics Canada (https://www.statcan.gc.ca/eng/rdc/index).

## Abbreviations

CCHS: Canadian Community Health Survey
CHMS: Canadian Health Measures Survey
CHSCY: Canadian Health Survey of Children and Youth
CI: confidence interval
GSS: General Social Survey
HBSC: Health Behaviour in School-Aged Children
ISAT: International Sedentary Assessment Tool
MEC: mobile examination centre
PASS: Physical Activity, Sedentary behaviour and Sleep
PHAC: Public Health Agency of Canada
TASST: Taxonomy for Self-reported Sedentary behaviour Tools
