# Supplementary material for: Sedentary behaviour surveillance in Canada: trends, challenges and lessons learned
Source: Int J Behav Nutr Phys Act. 2020 Mar 10;17:34. doi: 10.1186/s12966-020-00925-8 (PMC7063715; doi:10.1186/s12966-020-00925-8)
Supplement: Supplementary file 1 — Additional file 1: Table S1. Sedentary activities module uptake by region in the Canadian Community Health Survey. [file 12966_2020_925_MOESM1_ESM.docx]

**Supplemental table 1. Sedentary activities module uptake by region in the Canadian Community Health Survey**

| Year | Newfoundland | Prince Edward Island | Nova Scotia | New Brunswick | Ontario | Québec | Manitoba | Saskatchewan | Alberta | British Columbia | Yukon | Northwest Territories | Nunavut |
| --- | --- | --- | --- | --- | --- | --- | --- | --- | --- | --- | --- | --- | --- |
| 2000-01 |  |  |  | x | x | x | x | x |  |  | x |  |  |
| 2003 |  |  |  |  | x |  |  |  | x | x |  |  |  |
| 2005 |  |  |  |  |  |  |  | x |  |  |  |  |  |
| 2007-08 | x | x | x | x | x | x | x | x | x | x | x | x | x |
| 2009 | x |  |  |  |  |  | x |  |  | x |  |  |  |
| 2010 | x |  |  |  |  |  | x |  |  | x |  |  |  |
| 2011 | x | x | x | x | x | x | x | x | x | x | x | x | x |
| 2012 | x | x | x | x | x | x | x | x | x | x | x | x | x |
| 2013 |  | x |  |  |  |  |  |  |  |  |  |  |  |
| 2014 |  | x |  |  |  |  |  |  |  |  |  |  |  |
| 2015 |  | x |  |  | x |  |  |  |  | x |  |  |  |
| 2016 |  | x |  |  | x |  |  |  |  | x |  |  |  |
| 2017 | x | x | x | x | x | x | x | x | x | x | x | x | x |
| 2018 | x | x | x | x | x | x | x | x | x | x | x | x | x |
